# PD-L1 in plasmacytoid dendritic cells promote HBV persistence through disrupting humoral immune response

**DOI:** 10.3389/fimmu.2025.1545667

**Published:** 2025-04-24

**Authors:** Danyang Meng, Jinhao Wang, Lianqun Du, Xiaojun Hu, Ying Liu, Pengcheng Zhang, Jianjie Wang, Qingyang Dong

**Affiliations:** ^1^ School of Basic Medicine, Jiamusi University, Jiamusi, Heilongjiang, China; ^2^ Military Medical Sciences Academy, Academy of Military Sciences, Tianjin, China

**Keywords:** humoral immune deficiencies, pDCs, PD-L1, CHB, Tfh, GC B cell

## Abstract

**Objective:**

To investigate the efficacy of PD-L1 blockade in restoring humoral immune response against HBV.

**Methods:**

HBV‐persistent C57BL/6J mice were established through hydrodynamic tail vein injection of 10 µg pAAV‐HBV1.2 plasmid. Subsequently, mice treated i.p. with anti‐PD‐L1 and/or anti‐CTLA‐4 at specified time points, with dosages of 500 µg, 250 µg, and 250 µg, respectively. Additionally, 5 × 10^5^ magnetic bead‐purified plasmacytoid dendritic cells (pDCs) were adoptively transferred i.v. into the acute mouse model followed by anti-PD-L1 treatment. Quantitative real-time PCR was employed to assess the expression levels of costimulatory and tolerogenic molecules in two dendritic cell subsets. Serum HBsAg and HBsAb were measured using ELISA. Flow cytometry was utilized to quantify T follicular helper (Tfh) cells, regulatory T cells (Treg), and germinal center (GC) B cells.

**Results:**

PD-L1 blockade markedly enhanced the differentiation of Tfh cells and GC B cells in HBV-persistent C57BL/6J mice, thereby promoting HBV clearance. Additionally, pDCs exhibited an increased capacity to induce immune tolerance, with pDCs isolated from HBV carriers inducing viral persistence. This persistence was effectively counteracted by treatment with anti-PD-L1.

**Conclusion:**

pDCs mediate the dysregulation of the humoral immune response to HBV through PD-L1 in chronic hepatitis B infection, highlighting a promising target for the management of chronic HBV.

## Introduction

CHB (Chronic hepatitis B) infection remains a global health challenge, affecting approximately 256 million individuals and resulting in 1.1 million deaths annually, primarily from cirrhosis and hepatocellular carcinoma (1). Current therapies, including long-term nucleos(t)ide analogues (NUCs) and PEGylated interferon α (IFNα), are effective in suppressing HBV replication but rarely achieve a functional cure (2, 3). In the absence of treatment, 15–40% of individuals with chronic HBV infection progress to cirrhosis or develop liver cancer (4). Extended or indefinite treatment not only incurs significant costs but also limits accessibility, underscoring the urgent need for more effective and less toxic therapeutic strategies.

Recent advances in immunology have promoted the development of immunotherapeutic strategies targeting various diseases. In CHB patients, the immune landscape is characterized by an absence of neutralizing antibodies (5) and a deficiency of functional virus-induced immune cells response such as T cells (6), macrophages (7), natural killer (NK) cells (8), and dendritic cells (DC) (9). These deficits contribute to HBV-specific immune tolerance. Additionally, anti-HBs can directly bind viral particles and, with the aid of NK cells, destroy virus-infected cells through antibody-dependent cellular cytotoxicity (ADCC), thereby facilitating intracellular viral clearance (10).

Regulatory T cells (Tregs) inhibit T follicular helper (Tfh) cells differentiation via CTLA-4, disrupting humoral immunity and contributing to chronic HBV infection. Blocking of CTLA-4 can restore normal differentiation of Tfh cells and GC B cells, promoting HBV clearance (10, 11). Additionally, blocking PD-1 has been shown to enhance Tfh cell responses in seasonal influenza vaccination (12). Nevertheless, the role of PD-1/PD-L1 pathway in modulating dysregulated humoral responses in CHB infection warrants further investigation.

Our research focuses on the impact of PD-L1 blocking on HBV clearance and the restoration of humoral responses in CHB infection. Utilizing nontransgenic hydrodynamic transfection mouse models, we found that treatment with anti-PD-L1 significantly boosts Tfh-dependent humoral immune responses against hepatitis B surface antigens (HBsAg), thus promoting the elimination of HBV. This mechanism is critically dependent on pDCs, highlighting a novel therapeutic avenue in the management of chronic HBV infection.

## Results

### Acute and chronic HBV transfection mouse models

**Figure 1:**
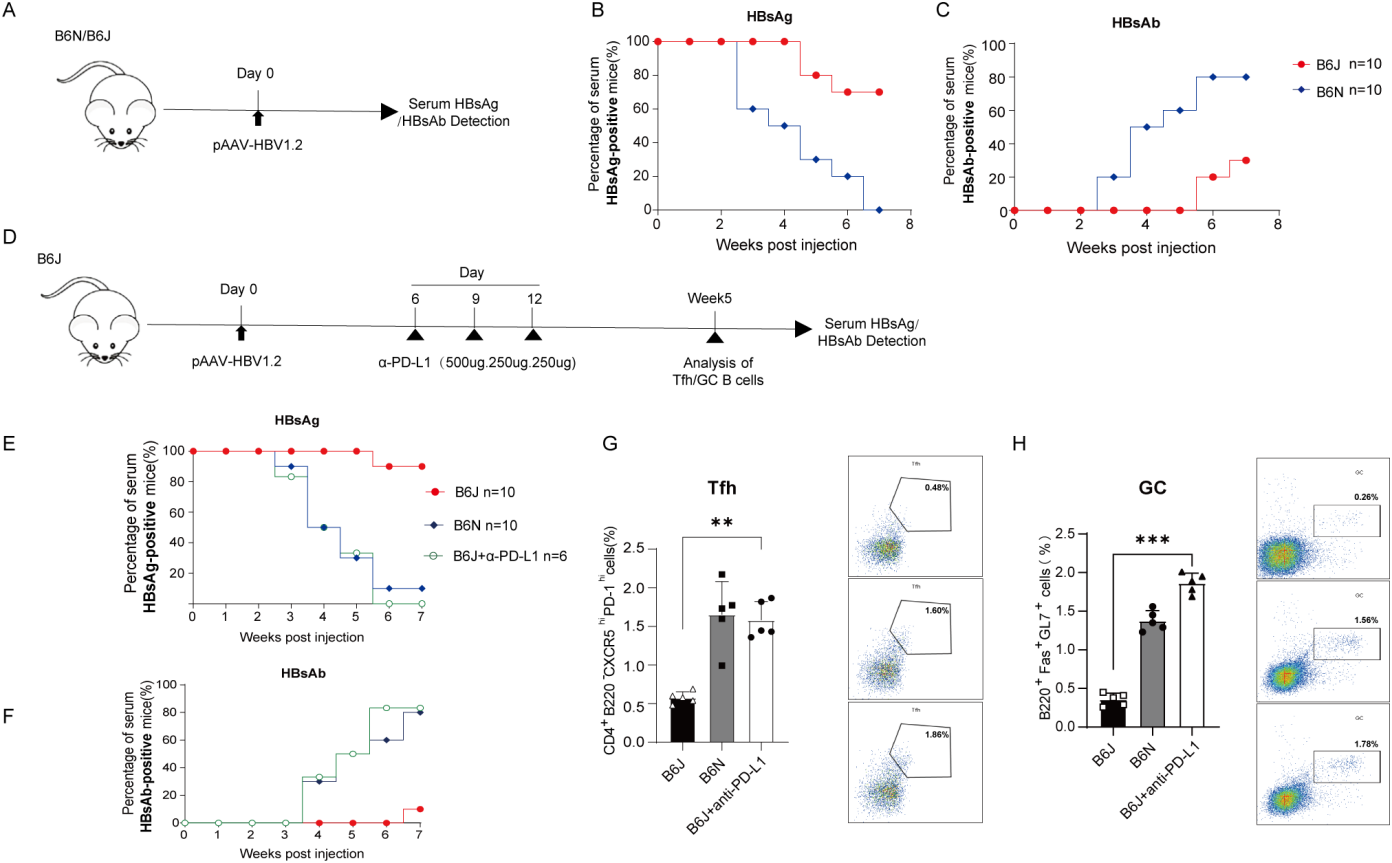
Enhanced HBV clearance in HBV persistent B6J mice treated with PD-L1 mAb. **(A)** Schematic representation of HBV transfection experiments in the study. **(B, C)** The positive rates of mice with detectable circulating HBsAg **(B)** and HBsAb **(C)** are shown. Data are from 10 mice and were replicated in at least two independent experiments. **(D)** Schematic representation of the PD-L1 monoclonal antibody (mAb) treatment experiments. B6J mice transfected with HBV received intraperitoneal injections of 500 µg PD-L1 mAb or an isotype control antibody on day 6 post-transfection. Subsequent administrations of 250 µg were performed on days 9 and 12. Serum samples were collected on a weekly basis, and splenic Tfh cells along with GC B cells were analyzed at the designated time points. **(E, F)** Evaluation of serum HBsAg and HBsAb via ELISA. Data are from 6–10 mice and were replicated in at least two independent experiments. **(G, H)** Percentages of splenic Tfh cells **(G)** and GC B cells **(H)** in B6J HBV-transfected mice treated with PD-L1mAb or isotype control were measured at week 5 post-injection, and untreated B6N transfection mice served as positive control. Data are from 5 mice and were replicated in at least two independent experiments. The data shown are mean ± s.e.m. Statistical analysis was performed using one-way Anova. **p < 0.01; ***p < 0.001.

In this study, we first established acute and chronic HBV transfection models in C57BL/6N and C57BL/6J respectively by administering 10 μg of the pAAV-HBV1.2 plasmid per mouse through hydrodynamic injection, as described previously (10) (**Figure 1A**). Serological analysis revealed that C57BL/6N mice exhibited a rapid decline in HBsAg positivity, coupled with a significantly higher positive rate of HBsAb compared to the B6J transfection model within 7 weeks post transfection (**Figures 1B, C**). These results mean that we successfully conducted chronic HBV transfected model in C57BL/6J.

### PD-L1 blockade promote viral clearance in HBV-persistent B6J mice

The PD-1/PD-L1 axis serves as a pivotal immune checkpoint, and blockade of this pathway has been shown to enhance humoral immune responses (13). Based on HBV-persistent B6J Mice, we assessed the impact of anti-PD-L1 treatment on viral clearance (**Figure 1D**). Serological analyses revealed a rapid decline in HBsAg positivity rates combined with enhanced HBsAb response following anti-PD-L1 treatment in HBV-persistent B6J mice, paralleling those observed in HBV transfected B6N mice (**Figures 1E, F**). Given that HBV transfection-induced humoral immune responses were previously established only in liver draining lymph nodes and spleen (10), we examined splenic Tfh (CD4^+^B220^-^CXCR5^hi^PD-1^hi^) and GC B (B220^+^GL7^+^Fas^+^) in HBV transfected mice via flow cytometry (**Supplementary Figure 1**). The results indicated that both Tfh and GC B cells underwent significant expansion following anti-PD-L1 treatment (**Figures 1G, H**). Collectively, these findings suggested that anti-PD-L1 therapy could restore humoral immune responses and facilitated the clearance of HBV in chronic HBV transfection mice.

### Blocking PD-L1 promote HBV clearance in HBV carrier mice

**Figure 2:**
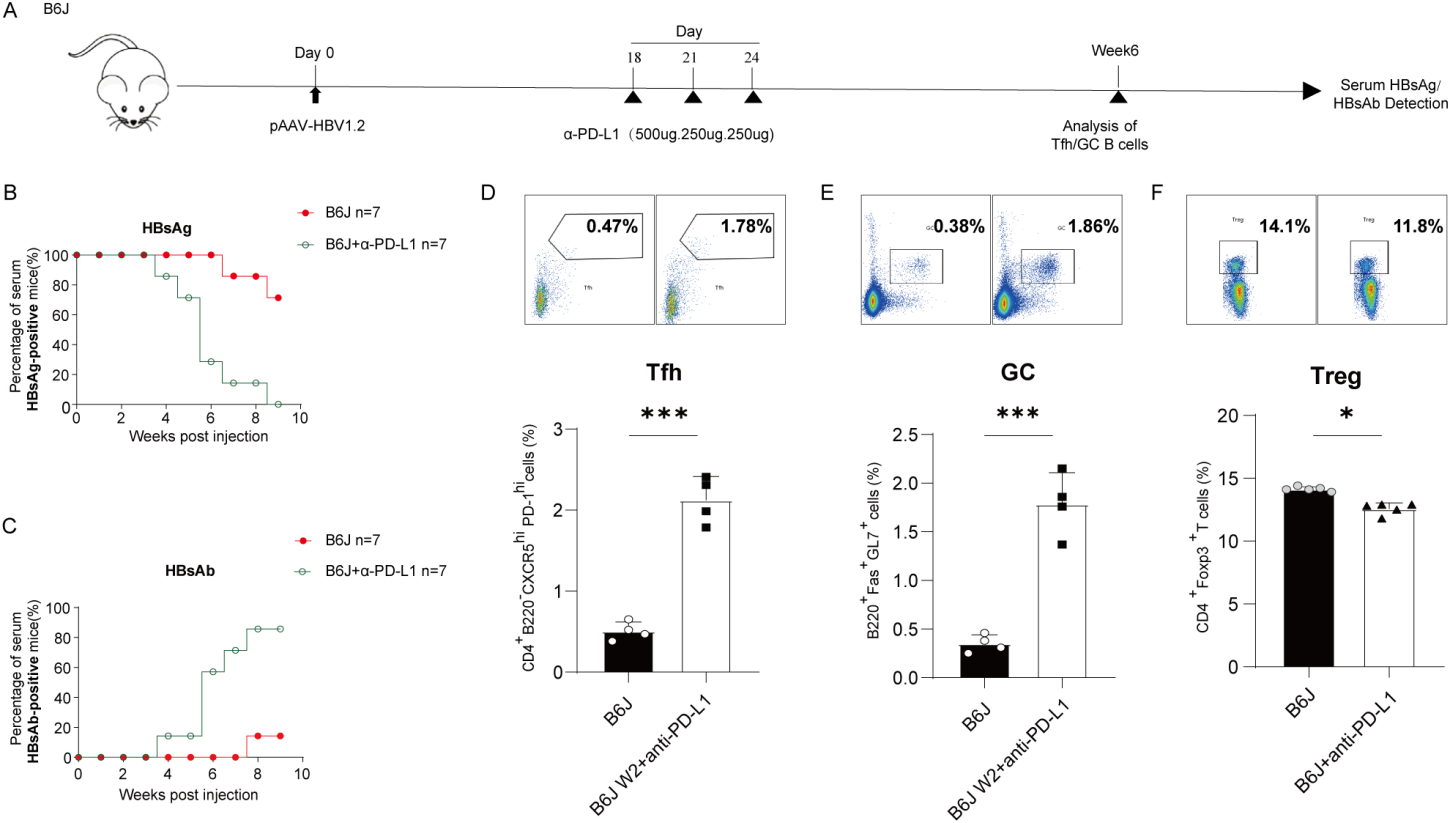
Treatment of PD-L1 mAb promote HBV clearance in HBV carrier mice. **(A)** Schematic representation of PD-L1 mAb treatment in the HBV carrier mice. Mice exhibiting high HBsAg detected by ELISA on day 18 post-transfection were classified as carriers and received treatment with PD-L1 mAb at indicated time course as described above. Serum samples were collected on a weekly basis, and proportions of splenic Tfh cells and GC B cells were analyzed three weeks post-treatment. **(B, C)** Positive rates of serum HBsAg and HBsAb were assessed via ELISA at designated times post-HBV transfection. Data are from 7 mice and were replicated in at least two independent experiments. **(D-F)** Percentages of splenic Tfh cells **(D)**, GC B cells **(E)**, and Treg **(F)** in HBV carrier mice treated with PD-L1mAb or isotype control were measured via flow cytometry three weeks post-treatment. Data are from 4–5 mice(D-E, n=4; F, n=5)and were replicated in at least two independent experiments. The data shown are mean ± s.e.m. Statistical analyses were performed using a two-tailed unpaired t-test. *p < 0.05; ***p < 0.001.

To evaluate the therapeutic efficacy of anti-PD-L1 in HBV carrier B6J mice, we employed C57BL/6J mice, establishing the model by administering the pAAV-HBV1.2 plasmid. On day 18 post-transfection (D18), mice with high HBsAg were identified as HBV carriers and subjected to anti-PD-L1 treatment (**Figure 2A**). Serological evaluations revealed that anti-PD-L1 treatment substantially promoted HBsAg clearance and HBsAb production (**Figures 2B, C**). Further analysis of humoral immune response indicated that differentiation of Tfh and GC B cells were restored following anti-PD-L1 treatment in HBV carriers (**Figures 2D, E**). Intriguingly, a significant reduction in Treg was also noted post-treatment (**Figure 2F** and **Supplementary Figure 2**). These findings collectively affirm that anti-PD-L1 therapy not only restore dysregulated anti-HBV humoral responses but also concurrently suppressed Treg differentiation in HBV carriers, highlighting its potential as a viable therapeutic strategy for managing chronic HBV infection.

### The therapeutic effect of anti-PD-L1 is independent of CD8^+^ T cells

**Figure 3:**
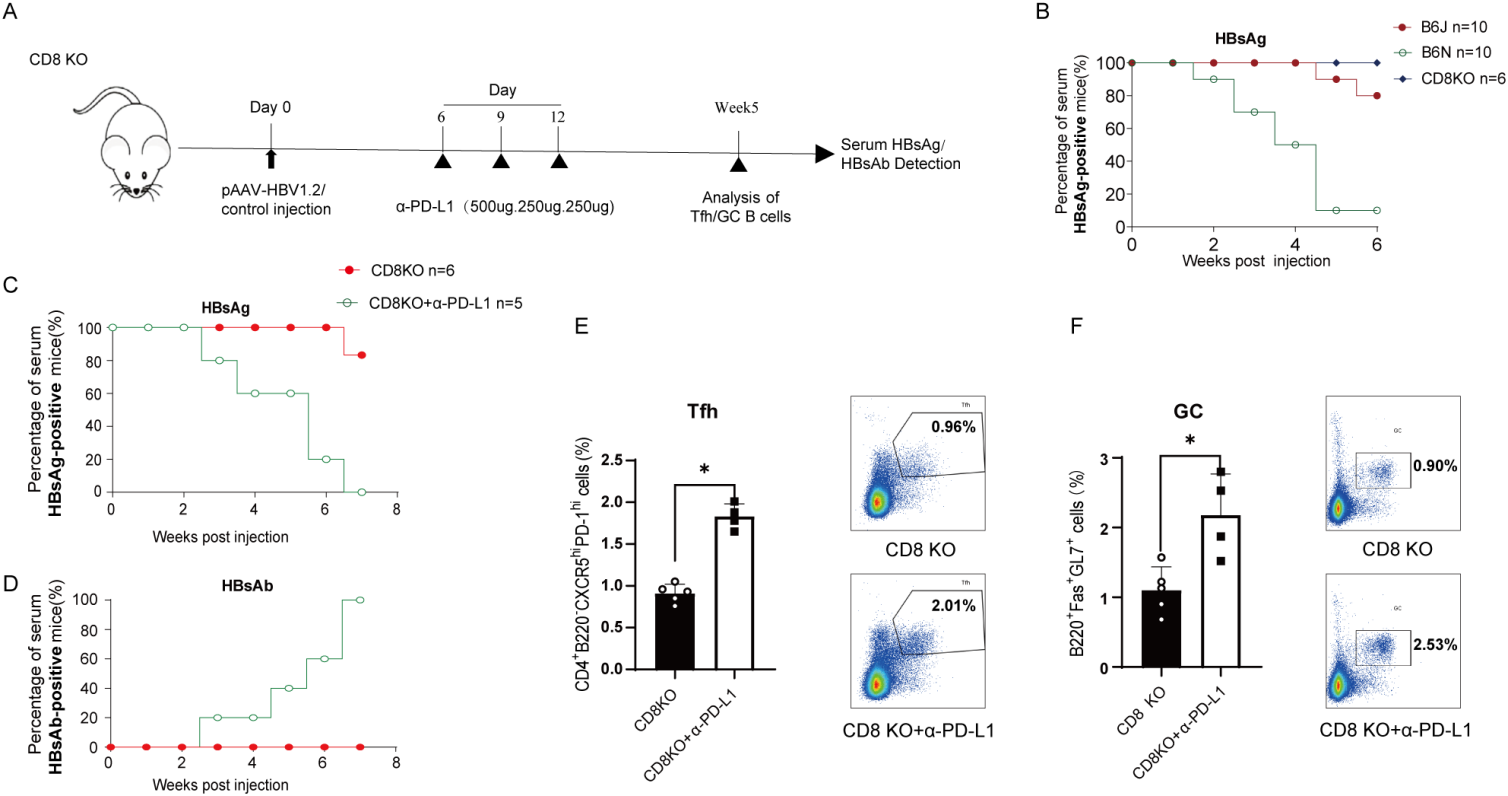
Enhancement of HBV clearance by PD-L1 mAb in CD8KO mice. **(A)** Schematic representation of the PD-L1 mAb treatment in CD8KO HBV transfection mice. CD8KO mice were hydrodynamically injected with 10 µg of the pAAV-HBV1.2 plasmid per mouse at day 0, and then treated intraperitoneally with 500 µg PD-L1 mAb or isotype control on day 6 post-transfection. Subsequent administrations of 250 µg were performed on days 9 and 12. Serum samples were collected on a weekly basis, and the proportions of splenic Tfh cells and GC B cells were analyzed at week 5. **(B)** Evaluation of serum HBsAg by ELISA at specified times post-HBV transfection in B6J, B6N, and CD8KO mice. Data are from 6–10 mice and were replicated in at least two independent experiments. **(C, D)** Positive rates of serum HBsAg and HBsAb were measured via ELISA. Data are from 5–6 mice and were replicated in at least two independent experiments. **(E, F)** Proportions of splenic Tfh cells **(E)** and GC B cells **(F)** in CD8KO HBV-transfected mice treated with PD-L1 mAb or isotype control were measured via flow cytometry 3 weeks post-treatment. Data are from 4–5 mice and were replicated in at least two independent experiments. The data shown are mean ± s.e.m. Statistical analysis was conducted using a two-tailed unpaired t-test. *p < 0.05.

In context of CHB pathogenesis, CD8^+^ T cell exhaustion plays a pivotal role, and treatment with anti-PD-L1 has been shown to alleviate this exhaustion, thereby promoting antigen clearance (14). To investigate whether the efficacy of anti-PD-L1 extends beyond the modulation of CD8^+^ T cell exhaustion, we first utilized CD8 knockout (CD8KO) mice to conduct HBV transfection and test of HBsAg showed significant HBV persistence without anti-PD-L1 treatment (**Figures 3A, B**). Then we treated CD8 KO transfected mice with anti-PD-L1 as described (**Figure 3A**), and serological evaluations indicated that, despite the absence of CD8^+^ T cells, anti-PD-L1 treatment significantly enhanced the clearance of HBsAg and production of HBsAb (**Figures 3C, D**). Concurrently, blockade of PD-L1 markedly augmented the differentiation of Tfh and GC B cells in CD8KO transfection mice (**Figures 3E, F**). Collectively, these findings demonstrate that the therapeutic effects of anti-PD-L1 on HBV-persistent mice are independent of CD8^+^T cells but are primarily mediated through restoration of humoral immune response to HBV.

### The efficacy of anti-PD-1 and/or anti-CTLA-4 on HBV clearance

**Figure 4:**
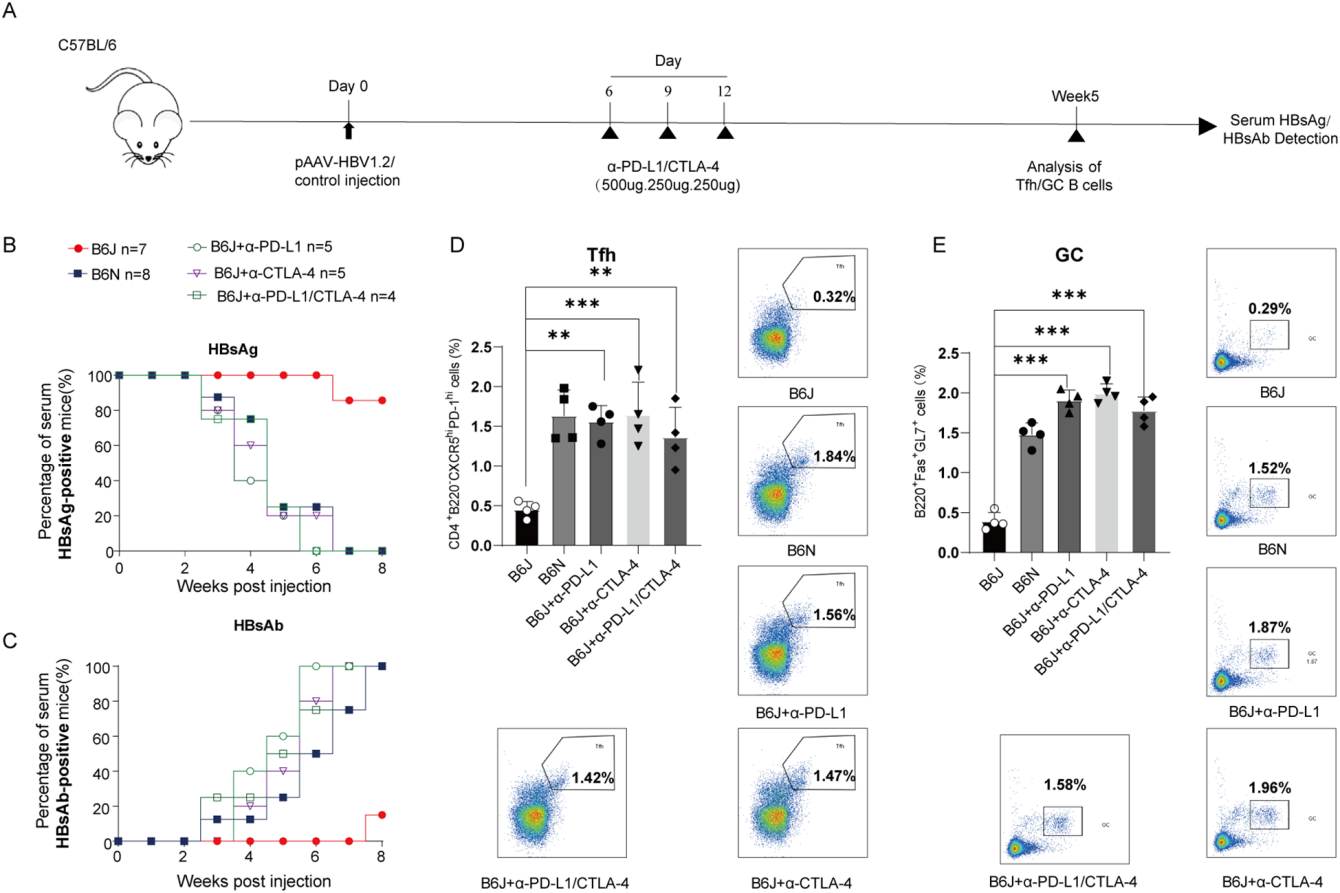
Combination therapy of CTLA-4 mAb and PD-L1 mAb. **(A)** Schematic representation of the combined CTLA-4 mAb and PD-L1 mAb treatment in B6J HBV persistent mice. B6J mice were hydrodynamically injected with 10 µg of the pAAV-HBV1.2 plasmid per mouse at day 0 and subsequently treated intraperitoneally with 500 µg of PD-L1 mAb and/or CTLA-4 mAb on day 6 post-transfection, followed by additional doses of 250 µg on days 9 and 12. Serum samples were collected weekly, and the proportions of Tfh cells and GC B cells were analyzed at week 5 post-injection. **(B, C)** Positive rates of serum HBsAg and HBsAb were measured via ELISA. Data are from 4–8 mice and were replicated in at least two independent experiments. **(D, E)** Percentages of splenic Tfh cells **(D)** and GC B cells **(E)** in B6J HBV persistent mice treated with PD-L1 mAb and/or CTLA-4 mAb, and untreated B6N transfection mice served as positive control. Data are from 4 mice and were replicated in at least two independent experiments. The data shown are mean ± s.e.m. Statistical analysis was performed using one-way Anova. **p < 0.01; ***p < 0.001.

Building on our previous findings that anti-CTLA-4 treatment facilitates HBV clearance by restoring dysregulated humoral immune responses in chronic HBV infection, we investigated the potential synergistic effects of dual blockade with anti-PD-L1 and anti-CTLA-4 (**Figure 4A**). Serological tests revealed no significant differences in the positivity rates of HBsAg and HBsAb between treatments with either anti-CTLA-4 or anti-PD-L1 alone, and combined therapy did not demonstrate a clear advantage over monotherapy (**Figures 4B, C**). Similarly, analyses of Tfh and GC B cell showed that combined therapy did not offer a clear benefit over monotherapy (**Figures 4D, E**). These results suggested that both agents may promote humoral immunity recovery via overlapping signaling pathways, with anti-PD-L1 potentially acting upstream of Treg.

### pDCs possess enhanced potential to drive immune tolerance

**Figure 5:**
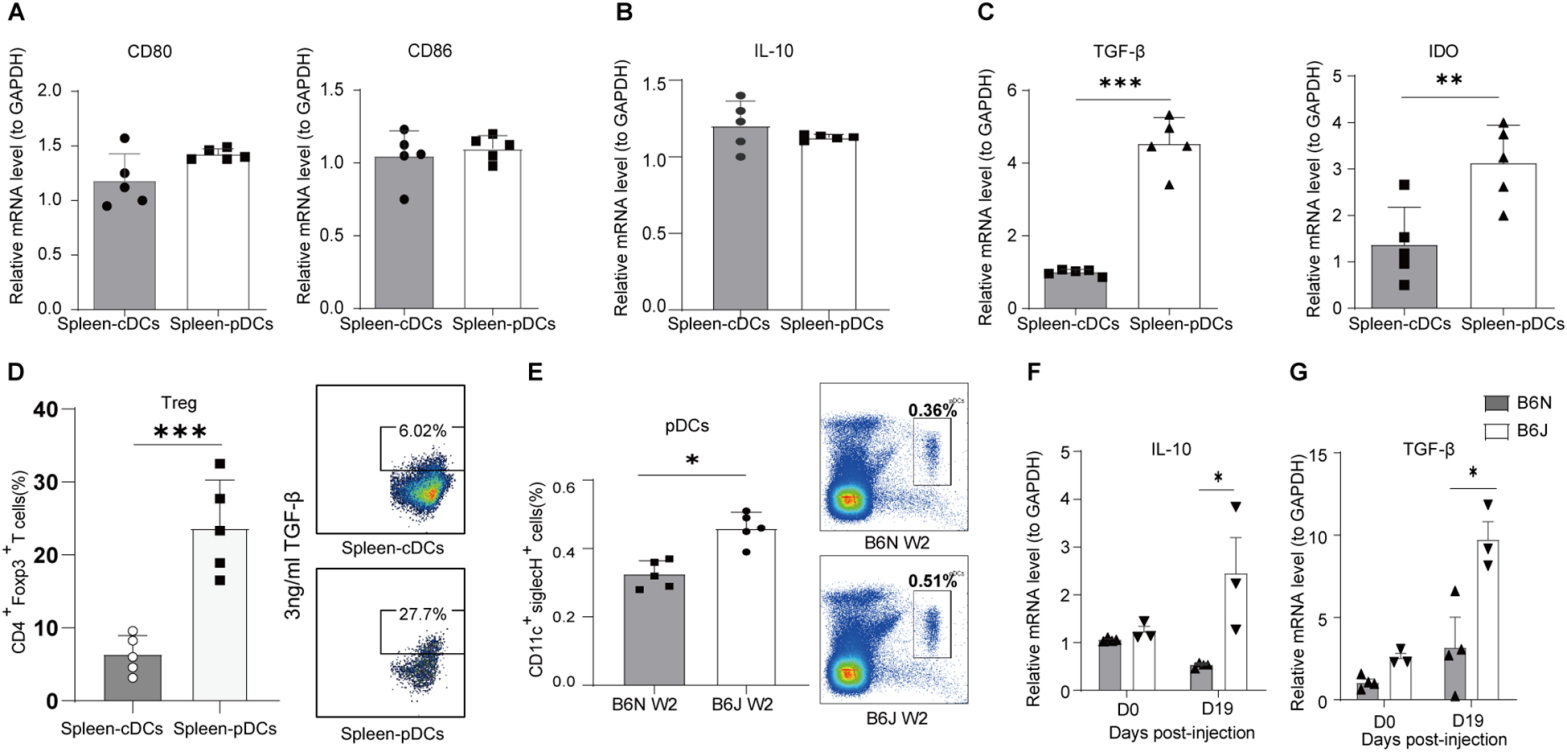
Differential expression of co-stimulatory and tolerogenic molecules in splenic cDCs and pDCs. Splenic pDCs and cDCs were sorted by BD FACSAria. **(A-C)** *CD80/86*, *IL-10*, *TGF-β and IDO* gene expression of splenic cDCs and pDCs cells from naïve B6J mice, Data are from 5 mice and were replicated in at least two independent experiments. **(D)** Percentage of Treg in DC-T co-culture Assay. Data are from 5 mice and were replicated in at least two independent experiments. **(E)** Percentage of splenic pDCs in B6N and B6J HBV transfection mice, measured two weeks post-injection, Data are from 5 mice and were replicated in at least two independent experiments. **(F, G)** IL-10 and TGF-β gene expression of splenic pDCs from B6J and B6N transfection mice at day 0 and days 19. Data are from 3–4 mice and were replicated in at least two independent experiments. The data shown are mean ± s.e.m. A 2-tailed unpaired t test was used to compare experimental groups. *p < 0.05, **p < 0.01, ***p < 0.001.

DCs, crucial antigen-presenting cells, include subtypes such as conventional DCs (cDCs) and plasmacytoid DCs (pDCs). We initially assessed the expression levels of costimulatory and tolerogenic molecules in splenic cDCs and pDCs of C57BL/6J mice. Our results revealed negligible differences in the expression of CD80/86 and IL-10 between the two DC subtypes (**Figures 5A, B**); however, pDCs exhibited enhanced expression of TGF-β and IDO (**Figure 5C**). Interestingly, this expression profile exhibited organ-specific variation, as hepatic pDCs showed selective upregulation of TGF-β without IDO elevation when compared to their cDCs (**Supplementary Figures S3A, B**). DC-T co-culture experiments demonstrated that splenic pDCs from B6J mice exhibited a significantly higher capacity to induce Treg differentiation compared to cDCs (**Figure 5D**), prompting further investigation into the role of splenic pDCs in HBV persistent mice. Subsequent analysis showed a higher proportion of splenic pDCs in the chronic HBV transfection mice compared to the acute model (**Figure 5E** and **Supplementary Figure 4**). Further examinations of IL-10 and TGF-β, which are critical for immune tolerance (15, 16), demonstrated that splenic pDCs from HBV carrier mice had a significantly greater capacity to express these molecules compare to B6N transfection mice (**Figures 5F, G**). Collectively, these results suggest that splenic pDCs in HBV persistent mice have enhanced ability to induce immune tolerance, highlighting their potential role in chronic HBV infection.

### Splenic pDCs from HBV persistent mice results in dysregulation of humoral immune response

**Figure 6:**
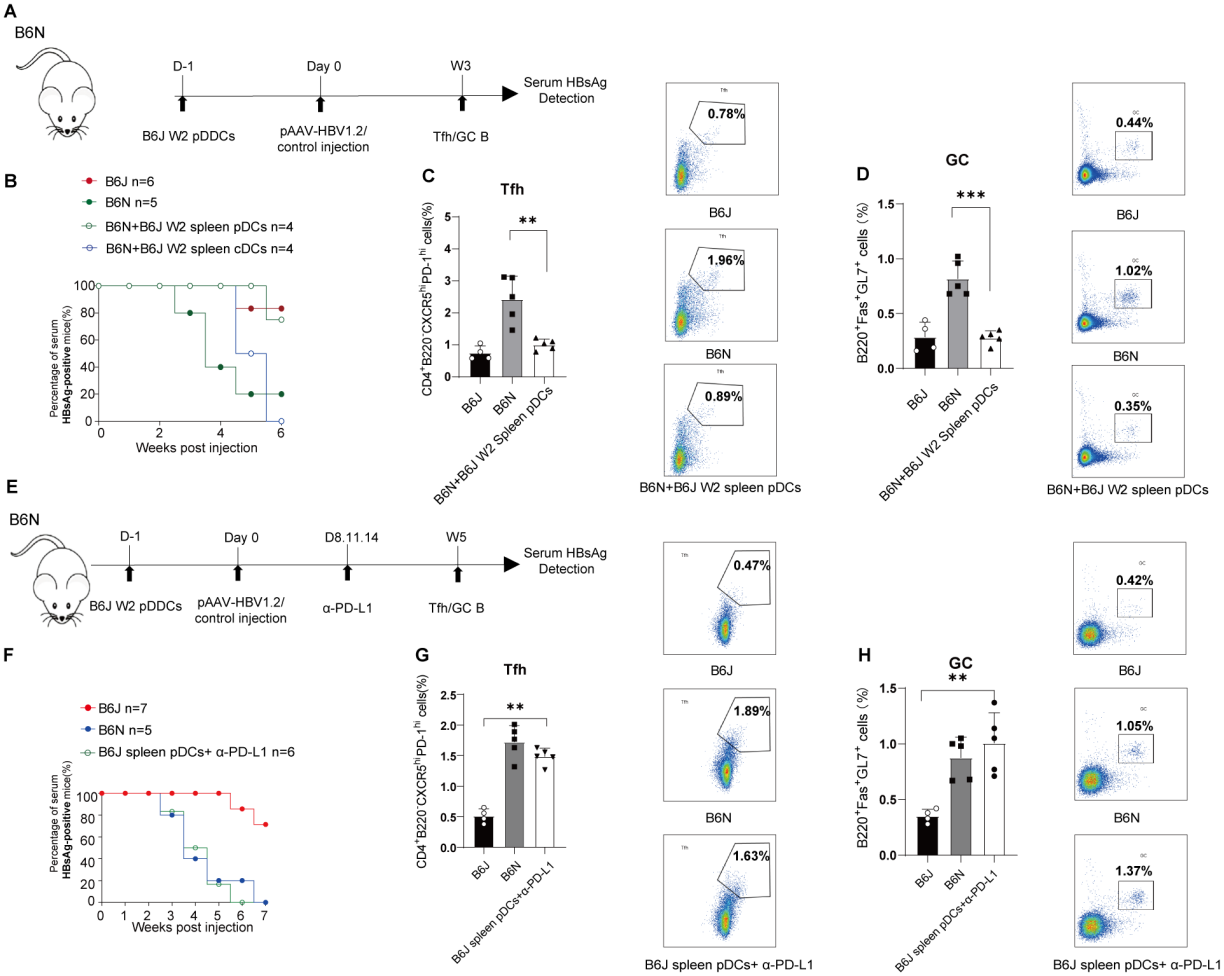
pDCs results in dysregulation of humoral immune response through PD-L1. **(A)** 5×10^5^ magnetic bead (MACS)-purified pDCs from B6J carriers were adoptively transferred to B6N which were challenged with 10ug pAAV-HBV1.2 plasmid 1 days later. B6N and B6J HBV transfection mice were conducted as control. Serum were collected weekly, and proportions of splenic Tfh cells and GC B cells were analyzed in three weeks post-injection. **(B)** Serum HBsAg examined by ELISA at specified times post-HBV transfection. Data are from 4–6 mice and were replicated in at least two independent experiments. **(C-D)** Frequencies of splenic Tfh cells **(C)** and GC B cells **(D)** of HBV-transfected recipients and control were measured three weeks post-injection. Data are from 4–5 mice and were replicated in at least two independent experiments. **(E)** 5×10^5^ MACS-purified splenic pDCs from B6J carriers were adoptively transferred to B6N which were challenged with 10ug pAAV-HBV1.2 plasmid 1 days later and subsequently treated with 500 µg PD-L1 mAb on day 8, followed by 250 µg PD-L1 mAb on days 11 and 14. Serum and splenic Tfh/GC B cells were analyzed at the indicated time. B6N and B6J HBV transfection mice were conducted as control. **(F)** Serum HBsAg measured by ELISA at specified times post-HBV transfection. Data are from 5–7 mice and were replicated in at least two independent experiments. **(G-H)** Frequencies of splenic Tfh cells **(G)** and GC B cells **(H)** in recipients and control were measured three weeks post-treatment. Data are from 4–5 mice and were replicated in at least two independent experiments. The data shown are mean ± s.e.m; statistical comparisons were made using one-way Anova. **p < 0.01, ***p < 0.001.

To elucidate the role of pDCs in the pathogenesis of chronic HBV infection, we conducted adoptive transfer of DCs. Initially, splenic pDCs or cDCs from the B6J carriers were transferred to B6N mice one day before HBV transfection as described (**Figure 6A**). The results indicate that, compared to splenic cDCs, pDCs significantly inhibit the clearance of HBsAg (**Figure 6B**). Additionally, transplantation outcomes for hepatic DC subpopulations show that neither pDCs nor cDCs from carriers inhibit the clearance of HBsAg (**Supplementary Figure 3C**). These findings suggest that splenic pDCs in carrier mice possess a marked ability to suppress HBsAg clearance. Subsequent experiments involving the transplantation of pDCs examined their impact on the humoral immune response, revealing that pDCs significantly inhibit the differentiation of Tfh and GC B cells (**Figures 6C, D**). To fully characterize the expression of co-stimulatory molecules in pDCs, we sorted splenic pDCs from B6J and B6N mice at D0 and W2 post transfection and analyzed the mRNA levels of CD80, CD86, and PD-L1. The results revealed no significant differences in these markers between groups, although PD-L1 mRNA showed an upward trend post-modeling (**Supplementary Figure 5A**). To further validate these findings, we conducted flow cytometry analysis to confirm the expression of PD-L1 on pDCs in the spleen. The results indicated PD-L1^+^ pDCs remained unchanged in acute HBV mice. However, in carrier mice, the frequency of PD-L1^+^pDCs was significantly elevated (**Supplementary Figures S5B, C**). Collectively, these results demonstrated that splenic pDCs significantly influence humoral immune responses and the clearance of HBV, underscoring their critical role in the dynamics of HBV infection.

### Anti-PD-L1 interventions counteract the suppressive impact of pDCs on humoral immune response

To further determine whether pDCs exert their suppressive effects via PD-L1, we transferred pDCs isolated from HBV carriers into B6N mice one day prior to HBV transfection, followed by interventions of anti–PD-L1 as described (**Figure 6E**). Serological analysis revealed that anti-PD-L1 effectively reversed the pDCs-mediated inhibition of HBV clearance (**Figure 6F**). Moreover, treatment with anti-PD-L1 restored the impaired differentiation of Tfh and GC B cells that result from the carriers’ pDCs (**Figures 6G, H**). Taken together, these findings delineate a mechanism that pDCs promote humoral immune dysregulation through the PD-1/PD-L1 axis in chronic HBV infection.

## Discussion

Investigations into immune checkpoints have elucidated mechanisms of immune tolerance prevalent in tumors and chronic diseases (17). Crucially, restoring defective immune response is a pivotal therapeutic strategy for HBV clearance in CHB patients. Previous studies have demonstrated that inhibition of CTLA-4 on Tregs enhances the humoral response in chronic HBV infection, thereby facilitating the elimination of HBV (10). Extending these findings, our research identifies the PD-L1 in pDCs as a critical contributor to humoral immune deficiency, likely through its role in promoting Treg differentiation. These insights significantly advance our understanding of humoral immune dysregulation in chronic HBV infection and propose novel therapeutic avenues for its management.

The dysfunction of cytotoxic T cells is widely recognized as a primary mechanism underlying chronic viral infection. Blocking of the PD-1/PD-L1 signaling pathway has been shown to reinvigorate exhausted CD8^+^ T cells in both tumor and chronic viral infections, thereby restoring their cytotoxic capabilities (12, 14, 18, 19). Additionally, PD-1 blocking has also been implicated in enhancing the humoral immune response (13, 20). In our studies conducted on a chronic HBV transfection mouse model, we demonstrate that treatment with anti-PD-L1 effectively restored the differentiation of Tfh and GC B cells to facilitate clearance of HBV, which is independent of CD8^+^T cell. Furthermore, the PD-1 pathway is integral to promoting Treg cell differentiation and sustaining immune tolerance (21). Our results further indicate that blocking PD-L1 inhibits Treg differentiation. The outcomes of combined treatment with anti-CTLA-4 and anti-PD-L1 suggest that both regulate the humoral immune response to HBV, implying that the restoration of humoral immunity in chronic HBV following PD-L1 blockade may be due to the inhibition of Treg differentiation.

Treg differentiation is influenced by various cellular interactions, with DCs playing a pivotal role in providing the necessary signals for Treg differentiation and survival (22, 23). A comparative analysis of tolerogenic molecules within two DC subsets revealed that pDCs express markedly higher levels of TGF-β and IDO than cDCs, suggesting that pDCs possess superior tolerogenic capabilities. In HBV-persistent B6J mice, the proportion of splenic pDCs was substantially higher than that of B6N acute mice, paralleling with more IL-10 and TGF-β, which are critical for immune tolerance. Adoptive transfer experiments demonstrated that splenic pDCs from HBV carriers hindered HBV clearance in the acute model. Importantly, co-treatment with anti-PD-L1 reversed the suppressive effect of pDCs from carrier mice on HBV clearance, suggesting that pDCs mediate inhibition of humoral immune response predominantly through the PD-1/PD-L1 pathway.

While our findings confirm that pDCs induced dysregulation of humoral immunity via PD-L1, resulting in HBV persistence, it is crucial to acknowledge that these results were derived using non-transgenic hydrodynamic transfection mouse models and comprehensive clinical studies are necessary to validate these observations. Future research should employ pDC-specific depletion models, such as BDCA2-DTR mice or anti-PDCA1 antibodies, to explore whether the removal of PD-L1^+^ pDCs can restore humoral immune responses and promote the clearance of HBsAg. Additionally, studies could investigate the antiviral capabilities of pDCs.

## Methods

### Mice

Male C57BL/6N (B6N) and C57BL/6J (B6J) mice were acquired from Charles River (Beijing), while CD8KO mice were kindly provided by Professor Lilin Ye. All mice were 6 weeks old and maintained at the SPF animal facility of the Tianjin Institute of Environmental and Operational Medicine. The Institutional Animal Care and Use Committee (IACUC) at this institute approved all experimental procedures involving mice.

### Hydrodynamic injection and serological test

Mice received hydrodynamic tail vein injections of 10 µg of the HBV expression plasmid pAAV-HBV1.2 (genotype A), a contribution from Dr. Pei-Jer Chen. Hydrodynamic tail vein injections has been described previously16. Serum samples were collected at designated intervals post-injection for the analysis of HBsAg and anti-HBs using ELISA kits as described (KeHua, Shanghai, China).

### Cut-off value (COV) determination

Negative Control (NC): Mean optical density (OD) of triplicate negative controls (NCx) was calculated as, NCx=(NC1 + NC2 + NC3)/3. If NCx < 0, it was set to 0. Positive Control (PC): OD value of positive control. Assay Validity Requirements: NCx ≤ 0.100;PC > 1.000. Blank control (for dual-wavelength reading) < 0.040; for single-wavelength reading ≤ 0.080. COV=NCx+0.100. Sample OD (S) and S/COV ratio were calculated. Positive: S/COV > 1.0;Negative: S/COV < 1.0.

### Lymphocyte isolation

Spleen tissues were disaggregated through a 70 µm nylon mesh (Falcon; BD Biosciences) using the rounded end of a 2 ml syringe plunger to achieve single cell suspensions. Spleen samples required further erythrocytes lysis using ACK buffer (R1010, Solarbio, China) and washed with stain buffer.

### Dendritic cell (DC) and T cell co-culture assay

CD4^+^CD25^-^ naïve T cells were purified from spleens of B6J mice via FACS. Sorted DCs (2.5×10^4^ cells/well) and naïve T cells (1.5× 10^5^ cells/well) were co-cultured in 96-well plates precoated with anti-CD3 and anti-CD28 mAbs. TGF-β (3ng/ml) were added to the cultures to induce Treg differentiation. On day 3 of co-culture, 50% of the medium was replaced, and 20 U/mL recombinant IL-2 (rIL-2) (PeproTech ^®^) was supplemented. After an additional 48 hours, cells were harvested, stained with CD4 (clone GK1.5) and Foxp3 (Invitrogen), and analyzed by flow cytometry.

### Flow cytometry

Following isolation, lymphocytes were stained at 4°C for 30 minutes with antibodies against CD16/32(clone 93), Fas (clone 15A7), PD-1(clone J43), GL-7(clone GL7) sourced from eBioscience. Antibodies against CD3(clone 145-2C11), CD4(clone GK1.5), CD11c (clone N418), SiglecH (clone551), B220(clone RA3-6B2), CD45(clone 30-F11) (clone M5/114.15.2) sourced from Biolegend.

### For Treg staining

For the labeling of Treg cells, Fixation/permeabilization kit (eBioscience) is used to membrane permeabilization. First, CD4 and CD16/32 is used for surface molecule labeling, then follow the reagent kit instructions for intracellular Foxp3 (Invitrogen)staining.

### For Tfh staining

The cells were first labeled with Biotin Rat Anti-Mouse CD185 (CXCR5) (BD Clone 2G8) and CD16/32 for 1.5 h at room temperature. Then the cells were washed with stain buffer and stained with PE Streptavidin (Invitrogen), CD4, B220 and PD-1.

A Celesta Cell Analyzer (BD Biosciences) was used for data acquisition, and analysis was conducted using FlowJo software.

### Antibody treatments

*In vivo* blockade of PD-L1 was performed using PD-L1 blocking antibodies (Clone 10F.9G2, BioXcell) or IgG, injected intraperitoneally at specified times after pAAV/HBV1.2 hydrodynamic injection. The initial dose was 500 ug per mouse, followed by two doses of 250 ug each.

*In vivo* blockade of CTLA-4 was performed using CTLA-4 blocking antibodies (Clone 9D9, BioXcell) or IgG, injected intraperitoneally at specified times after pAAV/HBV1.2 hydrodynamic injection. The initial dose was 500 ug per mouse, followed by two doses of 250 ug each.

### Real-time quantitative

RNA was extracted using RNeasy mini kits (Qiagen), treated with DNase I (Thermo Fisher Scientific), and reverse transcribed with the Revert Aid First Strand cDNA Synthesis Kit (Thermo Fisher Scientific). Gene expressions were quantified by an ABI Q5 using specific primers and normalized to GAPDH. The primers were as follows:

mGAPDH forward 5′-AGGTCGGTGTGAACGGATTTG-3′,
reverse5′ -TGTAGACCATGTAGTTGAGGTCA -3′;
mCD80 forward 5′-ACCCCCAACATAACTGAGTCT-3′,
reverse5′-TTCCAACCAAGAGAAGCGAGG-3′;
mCD86 forward 5′-TGTTTCCGTGGAGACGCAAG-3′,
reverse5′-TTGAGCCTTTGTAAATGGGCA-3′;
mIL-10 forward 5′-GCTCTTACTGACTGGCATGAG-3′,
reverse5′-CGCAGCTCTAGGAGCATGTG-3′;
mIDO forward 5′-GCTTTGCTCTACCACATCCAC-3′,
reverse5′-CAGGCGCTGTAACCTGTGT-3′;
mTGF-b forward 5′-TACCTGAACCCGTGTTGCTCTC-3′,
reverse5′-GTTGCTGAGGTATCGCCAGGAA-3′

### Adoptive-transfer study

pDCs were harvested from the spleens of designated mice utilizing a Plasmacytoid Dendritic Cell Isolation Kit (Miltenyi Biotec). A total of 5 × 10^5 pDCs were administered intravenously to recipient mice.

### Statistical analysis

Data analyses were conducted using Prism (GraphPad) employing t-tests (unpaired or paired as appropriate) or one-way ANOVA with significance thresholds set at *p < 0.05, **p < 0.01, and ***p < 0.001. Data are presented as mean ± s.e.m. The process of group assignment and subsequent evaluation of results was conducted under conditions of blindness to ensure impartiality. There were no exclusionary criteria set forth, and all collected samples were incorporated into the analysis without exception.

## Data Availability

The raw data supporting the conclusions of this article will be made available by the authors, without undue reservation.

## Supplementary material

Supplementary Figure 1 Gating strategies for Tfh and GC. **(A)** Gating strategy to determine the GC B cells in the spleen, using B220, GL-7 and FAS. **(B)** Gating strategy to determine the Tfh cells in the spleen, using B220, CD4, CXCR5 and PD-1.

Supplementary Figure 2 Gating strategy for Treg. Gating strategy to determine the Treg in the spleen, using CD3e, CD4 and Foxp3.

Supplementary Figure 3 Tolerogenic capability of liver cDCs and pDCs. **(A, B)** CD80/86, IL-10, TGF-β and IDO gene expression of liver cDCs and pDCs from naïve B6J mice. Data are from 3 mice and were replicated in at least two independent experiments. **(C)** Serum HBsAg examined by ELISA at specified times post-HBV transfection. Data are from 3–5 mice and were replicated in at least two independent experiments. The data shown are mean ± s.e.m. An unpaired t test was used to compare experimental groups, ***p < 0.001.

Supplementary Figure 4 Gating strategies for pDCs. Gating strategy to determine the pDCs in the spleen, using CD11c and SiglecH.

Supplementary Figure 5 Tolerogenic capability of splenic pDCs. **(A)** *CD80/86, CD86* and *PD-L1* gene expression of splenic pDCs from mice at D0 and W2 post transfection. Data are from 4 mice and were replicated in at least two independent experiments. **(B, C)** Percentage of PD-L1^+^pDCs in spleen of mice at D0 and W2 post transfection. Data are from 5–7 mice and were replicated in at least two independent experiments. The data shown are mean ± s.e.m. An unpaired t test was used to compare experimental groups, **p < 0.01.
