# Supplementary figures and images for: PD-L1 in plasmacytoid dendritic cells promote HBV persistence through disrupting humoral immune response
Source: Front Immunol. 2025 Apr 24;16:1545667. doi: 10.3389/fimmu.2025.1545667 (PMC12058763; doi:10.3389/fimmu.2025.1545667)

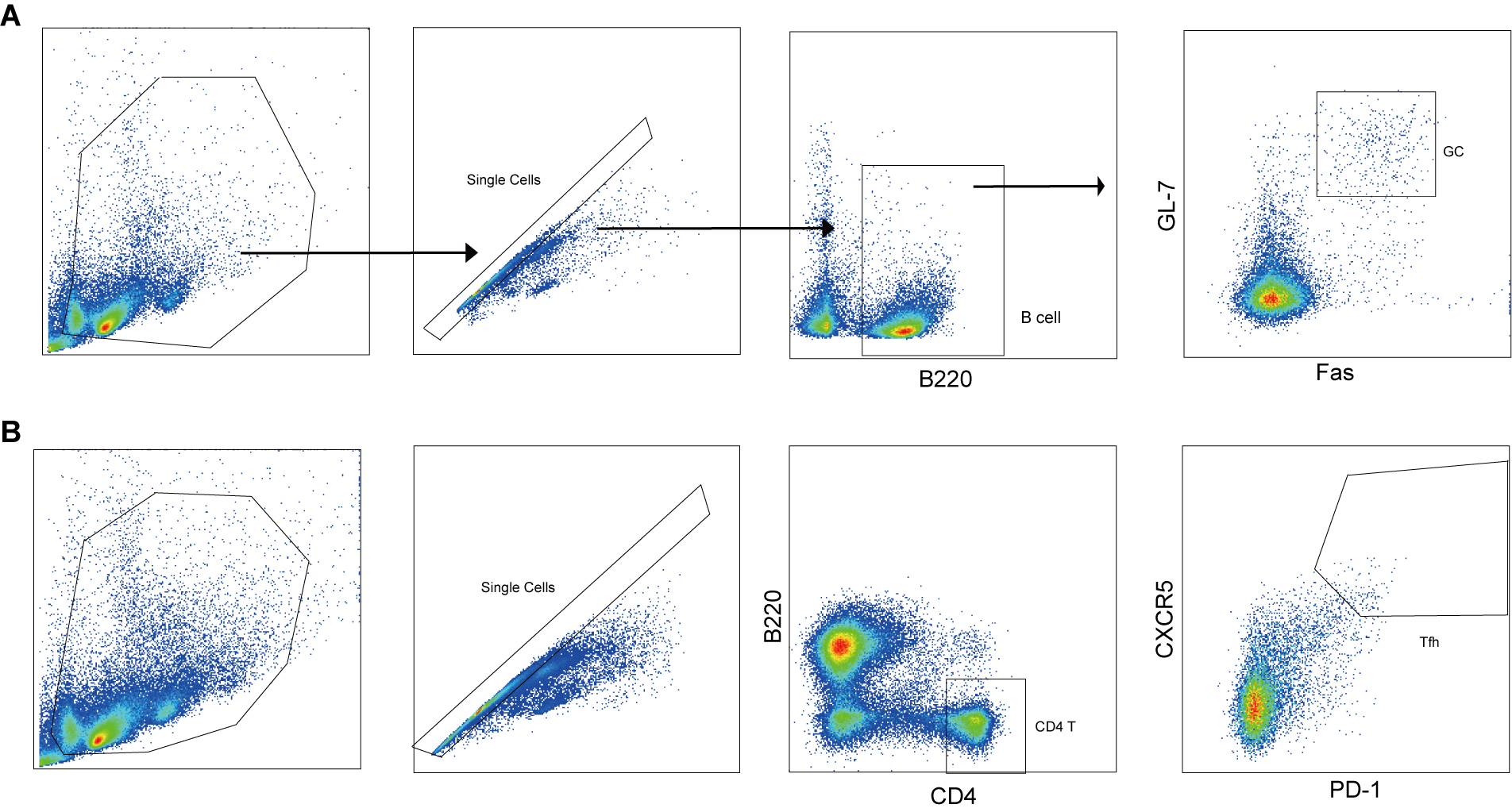

Supplement: Supplementary Figure 1 — Gating strategies for Tfh and GC. (A) Gating strategy to determine the GC B cells in the spleen, using B220, GL-7 and FAS. (B) Gating strategy to determine the Tfh cells in the spleen, using B220, CD4, CXCR5 and PD-1. [file Image1.tif]

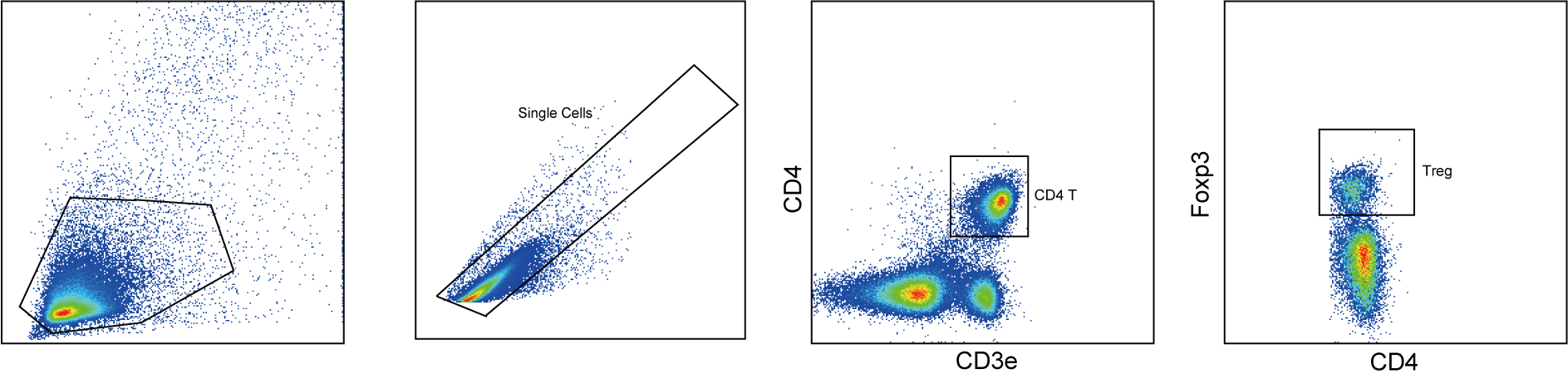

Supplement: Supplementary Figure 2 — Gating strategy for Treg. Gating strategy to determine the Treg in the spleen, using CD3e, CD4 and Foxp3. [file Image2.tif]

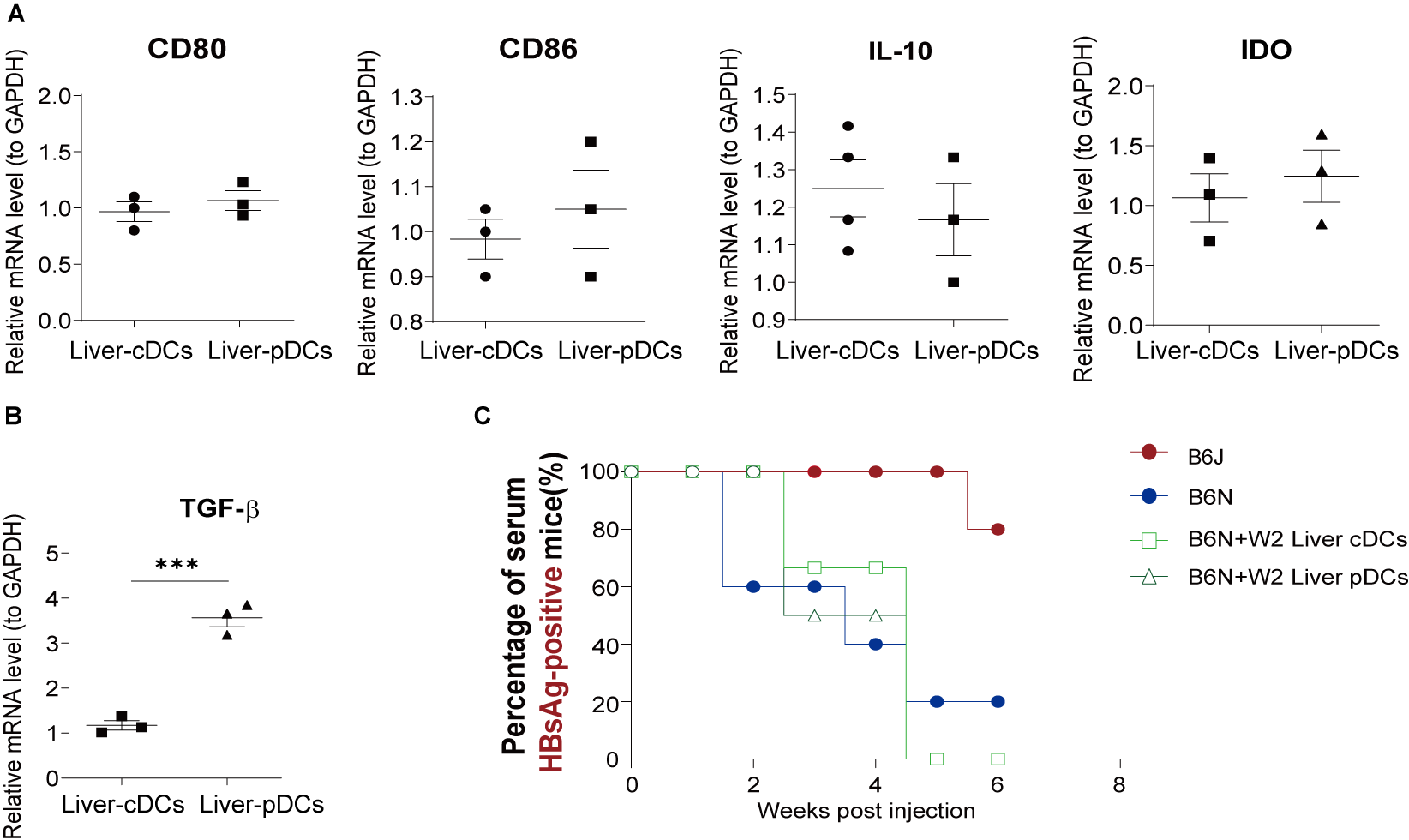

Supplement: Supplementary Figure 3 — Tolerogenic capability of liver cDCs and pDCs. (A, B) CD80/86, IL-10, TGF-β and IDO gene expression of liver cDCs and pDCs from naïve B6J mice. Data are from 3 mice and were replicated in at least two independent experiments. (C) Serum HBsAg examined by ELISA at specified times post-HBV transfection. Data are from 3–5 mice and were replicated in at least two independent experiments. The data shown are mean ± s.e.m. An unpaired t test was used to compare experimental groups, ***p < 0.001. [file Image3.tif]

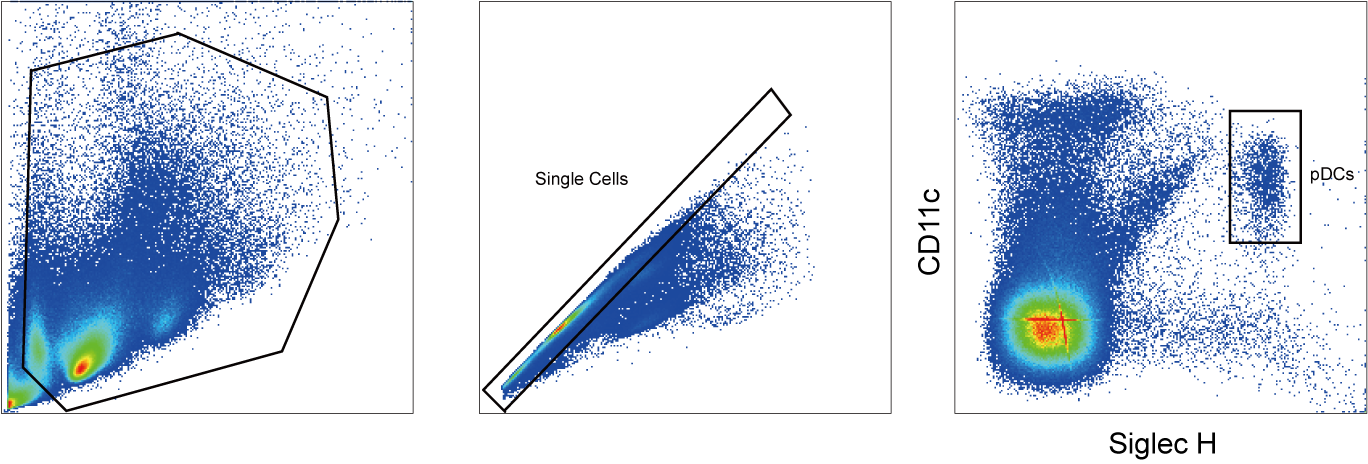

Supplement: Supplementary Figure 4 — Gating strategies for pDCs. Gating strategy to determine the pDCs in the spleen, using CD11c and SiglecH. [file Image4.tif]

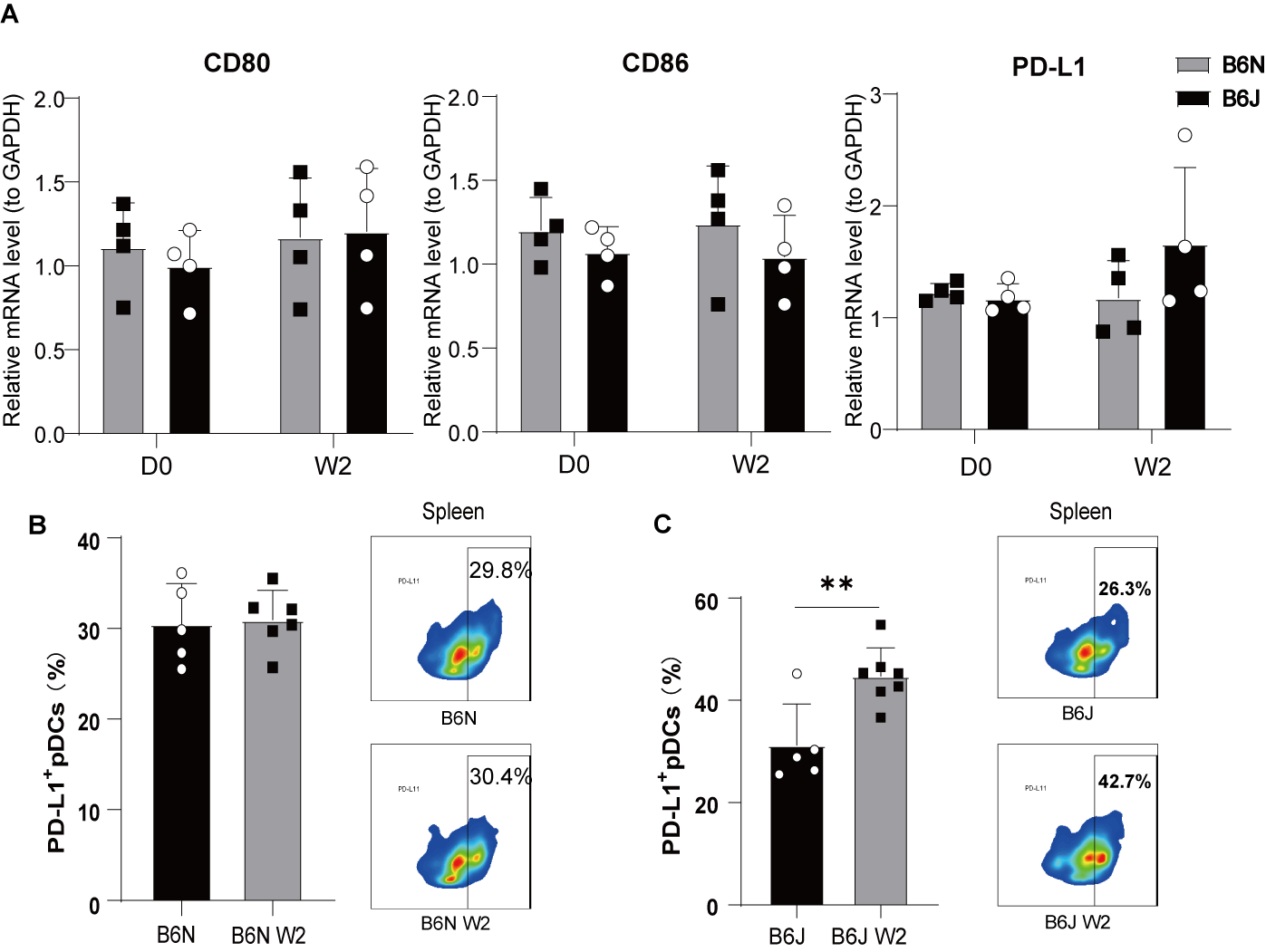

Supplement: Supplementary Figure 5 — Tolerogenic capability of splenic pDCs. (A) CD80/86, CD86 and PD-L1 gene expression of splenic pDCs from mice at D0 and W2 post transfection. Data are from 4 mice and were replicated in at least two independent experiments. (B, C) Percentage of PD-L1+pDCs in spleen of mice at D0 and W2 post transfection. Data are from 5–7 mice and were replicated in at least two independent experiments. The data shown are mean ± s.e.m. An unpaired t test was used to compare experimental groups, **p < 0.01. [file Image5.tif]
